# Clinical Variables Influence the Ability of miR-101, miR-150, and miR-21 to Predict Ventricular Remodeling after ST-Elevation Myocardial Infarction

**DOI:** 10.3390/biomedicines11102738

**Published:** 2023-10-10

**Authors:** Liana Maries, Alexandra Ioana Moatar, Maria Sala-Cirtog, Laurentiu Sima, Andrei Anghel, Catalin Marian, Aimee Rodica Chis, Ioan-Ovidiu Sirbu

**Affiliations:** 1 Biochemistry Department, “Victor Babes” University of Medicine and Pharmacy, 300041 Timisoara, Romania; maries.liana@umft.ro (L.M.); moatar.alexandra@umft.ro (A.I.M.); mariasala.cirtog@gmail.com (M.S.-C.); biochim@umft.ro (A.A.); cmarian@umft.ro (C.M.); ovidiu.sirbu@umft.ro (I.-O.S.); 2 Doctoral School, “Victor Babes” University of Medicine and Pharmacy, 300041 Timisoara, Romania; 3 Center for Complex Network Science, “Victor Babes” University of Medicine and Pharmacy, 300041 Timisoara, Romania; 4 Surgical Semiology Department, “Victor Babes” University of Medicine and Pharmacy, 300041 Timisoara, Romania; lica_sima@yahoo.com

**Keywords:** acute myocardial infarction, cardiac remodeling, microRNA, diabetes mellitus, hemoglobin

## Abstract

Left ventricle remodeling (LVR) after acute myocardial infarction (MI) leads to impairment of both systolic and diastolic function, a significant contributor to heart failure (HF). Despite extensive research in the field, predicting post-MI LVR and HF is still a challenge. Several circulant microRNAs have been proposed as LVR predictors; however, their clinical value is controversial. Here, we used real-time quantitative PCR to quantify the plasma levels of hsa-miR-101, hsa-miR-150, and hsa-miR-21 on the first day of hospital admission of MI patients with ST-elevation (STEMI). We analyzed their correlation to the patient’s clinical and paraclinical variables and evaluated their ability to discriminate between post-MI LVR and non-LVR. We show that, despite being excellent MI discriminators, none of these microRNAs can distinguish between LVR and non-LVR patients. Furthermore, we found that diabetes mellitus (DM), Hb level, and the number of erythrocytes significantly influence all three plasma microRNA levels. This suggests that plasma microRNAs’ diagnostic and prognostic value in STEMI patients should be reevaluated and interpreted in the context of associated pathologies.

## 1. Introduction

Coronary artery disease (CAD) is the leading cause of mortality and morbidity worldwide. The most common type of CAD is myocardial infarction (MI) [1]. Although the incidence of MI has decreased over the past years due to extensive nationwide prevention programs and emergency management guidelines, heart failure (HF) remains the most frequent complication of MI [2]. Multiple factors influence the development of HF post-MI, of which left ventricular remodeling (LVR) appears to be one of the most important [3]. Cardiac remodeling leads to impairment of the left ventricle’s systolic and diastolic functions; therefore, the need for biomarkers to predict this process is as critical as ever [4]. For the time being, various circulant biomarkers and imaging tools are available to identify and guide post-MI HF treatment. Of the circulant biomolecules, the following have been tested as markers of cardiac injury: cardiac troponins, CK and CK-MB, natriuretic peptides (N-terminal pro-brain natriuretic peptide), inflammation markers (C-reactive protein, neutrophil to lymphocyte ratio, and cytokines), renal biochemical markers (estimated glomerular filtration rate and cystatin C) and other biomarkers (matrix metalloproteinases, suppressors of tumorigenesis, galectin-3, and clusterin), but their accuracy is influenced by comorbidities and circadian variations in blood levels [5,6]. The imaging tools range from wall motion index score assessed by 2D echo, global longitudinal strain measured by speckle-tracking echocardiography, volumetric modifications assessed by 3D-echocardiography, myocardial velocities measured by Doppler tissue imaging, myocardial contrast echocardiography, stress echo, CMR-tissue tracking derived myocardial strain, or intramyocardial hemorrhage CMR detection [7,8,9,10,11,12]. While some of these methods have good prognostic potential, they are difficult to apply in practice due to a shallow learning curve, observer bias, and the necessity of complex instruments.

MicroRNAs (miRNAs) are short (21–24 nucleotides) RNA molecules that act as post-transcriptional regulators of gene expression in all aspects of cardiac pathogenesis, including LVR through modulation of both pro- and anti-fibrotic processes [13,14]. miRNAs influence both pro- and anti-fibrotic processes through the modulation of multiple targets in the transforming growth factor-beta signaling pathways. The proper, timely activation of a balanced miRNA post-MI response is instrumental for a favorable outcome.

Several circulant miRNAs have been proposed as post-MI LVR predictors. miR-101 protects against myocardial remodeling, and miR-150 attenuates cardiac remodeling and predicts post-MI HF [15,16,17]. miR-21 is a multifunctional microRNA with various roles in coronary heart disease. The data regarding miR-21 are inconclusive. While some studies show that miR-21 exhibits a cardioprotective role in MI by inhibiting cardiomyocyte apoptosis, other studies found that it contributes to cardiac remodeling fibrosis after MI [18,19,20,21,22,23,24,25]. However, clinical and animal data are conflicting, and their prognostic value concerning post-MI evolution is still highly controversial. Moreover, to the best of our knowledge, there is no systematic investigation of the association of these three circulant miRNAs to clinical and paraclinical parameters in MI patients.

The present study uses real-time quantitative PCR to investigate these circulant miRNAs in the clinical context of MI. It aims to characterize their association with the clinical and paraclinical variables, focusing on the ability of these miRNAs to predict post-MI ventricular remodeling. We show that none of these plasma microRNAs (as assessed on the day of admission) may serve as LVR predictors or correlate with biomarkers known for their variation after MI (TnI, CK, CK-MB, LDH, and ASAT).

## 2. Materials and Methods

### 2.1. Inclusion and Exclusion Criteria

This study has been conducted in accordance with the Declaration of the Helsinki Code of Ethics; the local hospital ethics review board has reviewed and approved the entire protocol (3822/31 May 2016). We enrolled (November 2016–October 2018) 105 patients diagnosed with first ST-elevation acute myocardial infarction (STEMI) at The Institute for Cardiovascular Disease in Timisoara, Romania. Each patient provided a signed, written consent. The protocol involved blood sampling and echocardiographic evaluations upon hospital admission. The echocardiographic studies were performed on a Philips machine in the intensive coronary care unit. We performed standard echo assessment from all commonly available views (parasternal long and short axis, apical 4 chambers, apical 2 chambers, apical 3 chambers, subcostal view). We assessed wall thickness in diastole, end-diastolic diameter of LV and RV, aortic annulus, left atrium, ascending aorta, systolic and diastolic function of LV (EDV, ESV, EF, E, and A wave) and RV, valvular stenosis or regurgitations, complications of MI (rupture of free wall, septum or papillary muscles), and presence of liquid. The Simpson method is the gold standard technique to evaluate ventricular volumes and ejection fraction and relies on tracing the endocardial border of the LV cavity in systole and diastole. Ejection fraction is calculated with the formula EF(%) = [(EDV − ESV) × 100]/EDV [26]. Only 43 patients participated in the follow-up echocardiographic evaluation one-year post-STEMI. The same method was used to appreciate the EF in the follow-up lot.

The inclusion criteria are:

- STEMI diagnostic based on the Third Universal Definition of Myocardial Infarction guidelines issued by the European Society of Cardiology (ESC) in 2012 [27];
- Hospital admission within 12 h from the onset of MI symptoms;
- Pre-hospital care according to the ESC guidelines for STEMI (antiplatelet: Aspirin 300 mg, Ticagrelor 180 mg or Clopidogrel 600 mg, Atorvastatin 80 mg, and fibrinolytic therapy and anticoagulation in selected patients);
- Age over 18.

The exclusion criteria are:

- History of CAD;
- Cardiac arrest resuscitated before hospital admission;
- Valvular disease (moderate/severe stenosis or regurgitation);
- Associated diagnostics of cancer, acute infectious diseases, and liver dysfunction;
- Inability to provide signed, written consent.

All patients underwent primary percutaneous coronary intervention (PCI), routine early PCI, or rescue PCI after fibrinolysis, according to the ESC guidelines.

Since the measurement of left ventricular longitudinal strain is not yet accessible in our emergency room (ER) unit, LVR was diagnosed when a minimum of a 10% increase from the baseline end-diastolic volume (ΔEDV) was documented at the follow-up evaluation. The follow-up lot was stratified into the LVR group (the patients who developed LV dysfunction and HF symptoms (NYHA class 2 or higher), LVEF < 50%; ΔEDV ≥ 10%, n = 14) and the non-LVR group (the patients who did not develop LV dysfunction and HF symptoms (NYHA class I), LVEF ≥ 50%; ΔEDV < 10%; n = 29).

The control group consists of 17 patients without any medical history or clinical signs of MI, for which all the exclusion criteria apply.

### 2.2. Specimen Collection

The blood samples (3 mL) were collected in EDTA-coated tubes and processed within 10 min for plasma separation (10 min centrifugation at 1500 rpm and room temperature). The plasma was collected and stored at −80 °C until further use. Plasma samples showing signs of hemolysis, hyperlipemia, and icterus were discarded.

### 2.3. RNA Purification

Total RNA was purified from 200 µL of plasma using a miRNeasy Serum/Plasma Kit (Qiagen, Hilden, Germany, catalog no. 217184), according to manufacturer instructions. RNA quality and quantity (A260/A280 and A230/260 ratios) were evaluated spectrophotometrically using a Nanodrop 2000 instrument.

### 2.4. PCR Detection

All RNA samples were spiked in with *Caenorhabditis elegans* cel-miR-39. We used the TaqMan^®^ MicroRNA Reverse Transcription Kit (Applied Biosystems, Waltham, MA, USA, catalog no. 4366596) for reverse transcription starting from the equal (10 ng) total RNA input. All qRT-PCR reactions were performed in duplicate, using inventoried TaqMan™ MicroRNA Assays (ThermoFisher Scientific, Waltham, MA, USA, assays ID 000438, 000473, 000397, 000200). Fold changes were calculated using the ΔΔCT method of relative quantification with *C. elegans* miR-39 as a normalizer [28].

### 2.5. Statistical Analysis

All statistical analyses were performed on Prism 9 for MacOS, Version 9.3.1. We used basic descriptive statistics to describe the demographic, clinical, functional, and laboratory data of the patients. Data distribution was checked using the Kolmogorov–Smirnov test; the heteroscedastic Student’s *t*-test (for variables with normal distribution) and Mann–Whitney U test (for data not normally distributed) were used to assess the differences between continuous variables. We used the Z-test to compare the binary variables datasets. All correlation analyses were performed using the Spearman test; all ROC/AUC analyses were performed using the Wilson/Brown method for 95% CI calculation. For all tests, the threshold of statistical significance is 0.05. All statistical tests are two-tailed.

## 3. Results

### 3.1. Baseline Clinical Data of Patients

Table 1 presents the demographic features and clinical data for the 105 STEMI patients. The cohort’s median age was 61 (ranging from 29 to 87) years, and female patients represented 27.62%. In terms of risk factors, the most frequent ones were hypertension (71.43%), smoking (50.48%), and obesity (29.52%). The median time interval between the onset of symptoms and reperfusion was 6 (1.5–16) hours; seven patients (6.67%) died in the hospital. All data can be accessed in Supplementary Table S1.

Only 43 patients had an echocardiographic evaluation one year after the STEMI and were, thus, included in the follow-up group. Table 2 shows the demographic, clinical, and echocardiographic parameters of LV function upon admission and at follow-up, stratified by the presence or absence of remodeling. Compared to non-LVR, LVR patients showed significantly different changes in ejection fraction and EDV volumes at discharge from the hospital and the follow-up. The prevalence of risk factors and the location of STEMI were similar between patients with and without LVR. All follow-up data can be accessed in Supplementary Table S2.

### 3.2. miRNA in STEMI Patients vs. Controls

Compared to the control group, the plasma levels of all three miRNAs (miR-101, miR-150, and miR-21) were significantly increased in patients with STEMI. The receiver operating characteristic (ROC) curve analysis showed areas under the ROC curve (AUC) ranging from 0.78 (miR-150) to 0.885 (miR-21), confirming that these miRNAs could be used as diagnostic biomarkers for STEMI (Table 3). Of note, there are no significant differences between plasma levels of any of the miRNAs in males vs. females.

### 3.3. miRNA and Clinical Parameters in STEMI Patients

We have systematically evaluated the correlation of the three miRNAs with the clinical and paraclinical parameters accessible through the electronic hospital archive (Table 4 and Supplementary Table S1). None of the three miRNAs correlates with any of the biomarkers known for their variation in STEMI (troponin I, CK, CK-MB, LDH, and ASAT). All three miRNAs display a statistically significant correlation to diabetus mellitus (DM) and, except for plasma miR-21, to erythrocyte/hematocrit and hemoglobin levels (Table 4). miR-150 also correlates marginally to age (Spearman coefficient r = 0.24; p value = 0.012), obesity (r = 0.272; *p* = 0.006), creatinine level (r = 0.312; *p* = 0.001), lymphocytes (r = −0.263; *p* = 0.007), and erythrocyte sedimentation rate (r = −0.196; *p* = 0.047). Of note, there is a strong correlation between all three plasma miRNAs, presumably reflecting a common tissue source and/or a common physio-pathological regulatory mechanism.

### 3.4. miR and Hb Levels

Given the well-known impact of Hb on the clinical outcome of MI, we further stratified our cohort according to Hb level: Hb < 13 g/dL, between 13 and 15 g/dL, and over 15 g/dL [16]. All miRNAs show gradual increases in both FC values and MI discriminative power with Hb level (Table 5). Interestingly, the correlation to DM has been preserved for the miR-150 group (Spearman coefficient r = 0.568; *p*-value = 0.014), miR-101 (r = 0.477; *p* = 0.045), miR-21 (r = 0.2715; *p* = 0.038) in the Hb 13–15 g/dL group, and only for miR-150 (r = 0.363; *p* = 0.049) in the Hb > 15 g/dL group. Furthermore, in the Hb 13–15 g/dL group, miR-101 (r = 0.318; *p* = 0.035) and miR-21 (r = 0.349; *p* = 0.020) correlate to the time elapsed since the onset of symptoms. The correlations between all three plasma miRNA levels are preserved except for miR-150 and miR-101 in the Hb > 15 g/dL group. None of the STEMI biomarkers (troponin I, CK, CK-MB, LDH, and ASAT) correlate with any of the three miRNAs in any of the three Hb subgroups.

### 3.5. miRNA in LVR vs. Non-LVR Group

The expression levels of plasma miRNAs are not significantly different in the LVR vs. non-LVR groups, and their AUCs are modest and statistically insignificant (Table 6). Furthermore, none of the miRNAs are correlated with the changes in EF and EDV in the LVR group, while in non-LVR patients, all three miRNAs are (negatively) correlated with the change in EF.

The correlation to DM is lost for all miRNAs; none of the miRNAs in either group are associated with Hb levels, erythrocyte numbers, or hematocrit levels.

There is a strong correlation between all three plasma miRNAs in the non-LVR group, while in the LVR group, miR-150 becomes disconnected from the other two miRNAs. In the LVR group, all correlations to DM, Hb level, number of erythrocytes, and hematocrit are lost; smoking is correlated to miR-21 (r *=* 0.587; *p =* 0.034); the time elapsed since the onset of symptoms is negatively correlated to miR-101 (r = −0.689; *p =* 0.032) and miR-21 (r = −0.659; *p =* 0.043).

### 3.6. miRNA in Diabetic vs. Non-Diabetic Patients

In our study, demographics, cardiovascular risk factors, and type of myocardial infarction are not significantly different between diabetic and non-diabetic patients (Table 7). Nevertheless, significant differences in terms of blood hypertension (*p* = 0.048) and in-hospital death ratios (*p* = 0.0015) in favor of diabetic patients have been noticed.

All three plasma miRNA levels are significantly downregulated in diabetic vs. non-diabetic MI patients; their ability to discern between diabetic and non-diabetic MI patients is rather modest (Table 8). Of note, the diabetic status strongly influences the ability of all three miRNAs to differentiate between MI and controls.

The miRNAs’ correlations to erythrocytes, Hb levels, and hematocrit are partially preserved in diabetic and non-diabetic subgroups. In the non-diabetic cohort, miR-101 is negatively correlated to erythrocytes (r = −0.104; *p* = 0.004) and hematocrit (r = −0.16; *p* = 0.02), while miR-150 is positively correlated to obesity (r = 0.285; *p* = 0.011). In the diabetic subgroup, except for miR-21, the other two miRNAs have preserved their negative association with Hb levels.

In non-diabetes patients, miR-150 correlates to miR-101 and miR-101 to miR-21, and in the diabetic cohort, only miR-21 is correlated to the other two miRNAs.

## 4. Discussion

The role of plasma miRNAs as diagnostic and prognostic markers post-MI is still disputed, mainly due to methodological differences in defining post-MI adverse events (like LVR), the timing of blood sampling, and the analytical platforms used. Here, we analyzed the early (hospital admission day) plasma expression level of three miRNAs (miR-101, miR-150, and miR-21), presumed LVR prognostic biomarkers, with a special focus on diabetes and hemoglobin levels.

### 4.1. miRNAs and Hb Level

All three miRNAs negatively correlate to Hb levels, the number of erythrocytes, and the hematocrit value. Of note, to our knowledge, none of the three miRNAs are expressed in erythrocytes; hence, none are expected to increase upon hemolysis.

Interestingly, the MI discriminative power of all miRNAs is strongly impacted by the Hb level: the lower the Hb, the more modest and less statistically significant the AUC. In the absence of data relating PaO_2_ to miRNA levels in these patients, one can only speculate that this might be due to better tissue (myocardial included) oxygenation. The relationship of these miRNAs to Hb and the number of erythrocytes and the hematocrit is interesting since both animal and human studies have shown that plasma Hb level impacts the clinical outcome of MI, and anemia is a risk factor in post-MI evolution [29,30,31,32,33,34].

miR-21 is hypoxia-responsive and shows significant cardioprotective effects against hypoxia lesions upon in vivo and ex vivo overexpression [21,35,36]. A significant hematocrit/Hb-related decrease in serum miR-21 levels was recently reported in a small cohort of chronic HF patients with reduced EF [37]. Ex vivo and in vivo experiments identified miR-21 as a major regulator of hematopoiesis through the SMAD7/TGF-beta signaling axis, and the inhibition of miR-21 increased the hematocrit level [38].

Plasma miR-150 (but not miR-21) was found to be significantly upregulated in aplastic anemia patients; this is in line with our data showing a negative correlation with the level of Hb and the number of erythrocytes. Of note, miR-150 was proposed as a clinical evolution biomarker in aplastic anemia, being (together with miR-1) an excellent responder to immunosuppressive therapy [39]. The negative correlation of miR-150 with Hb (and possibly chronic hypoxia) is in line with its demonstrated cardioprotective protective effect in vitro [40,41,42,43].

### 4.2. miRNAs and DM

DM strongly influences LV dynamics (although not necessarily through LVR) in diabetic patients with HF and MI, with a significant impact on life quality and cardiovascular outcome [44,45]. Early post-MI glycated hemoglobin value correlated negatively with the EF and predicted worse EF [46]. In our MI cohort, 24 patients have diabetes, and the levels of all three miRNAs are significantly decreased compared to non-diabetic MI patients. The diabetic status per se impacts the ability of these miRNAs to discern between MI patients and healthy controls, although with no effect regarding the relationship to LVR.

miR-21 has long been associated with DM and its complications (diabetic retinopathy, neuropathy, and nephropathy) [47]. miR-21 is downregulated in the plasma of diabetic patients and might signal diabetic cardiomyopathy patients [48,49].

Circulant miR-101 level is altered in diabetic patients and has been proposed as a diagnostic biomarker for type I and type II DM [50,51]. Together with six other miRNAs, miR-101 predicts type II DM remission in patients with CAD from the CARDIOPREV study [52].

The relationship of miR-150 to DM has been more thoroughly investigated. Circulant miR-150 is significantly decreased in both type I and II DM patients, being associated with islet autoimmunity and (together with miR-21) diabetic retinopathy [48,53,54]. The significance of miR-150 association with both MI and diabetes is underlined by a recent communication highlighting the role of miR-150 in the modulation of GLUT4 and glucose utilization in rat cardiomyocytes [55]. Equally significant, circulant miR-150 (together with five other miRNAs) predicts DM development in patients with CAD from the CORDIOPREV Study [56].

### 4.3. miRNAs and LVR

LVR refers to changes in ventricular size, shape, and function. It is a consequence of fibrosis, cardiomyocyte hypertrophy, and apoptosis. Fibrosis is the main component of post-MI remodeling and represents an unrestrained accumulation of extracellular matrix (ECM) components produced mainly by fibroblasts [57]. This leads to the organization of a permanent scar and a loss of function in the affected area. LVR is initiated in the acute phase of MI when transforming growth factor-beta (TGF-β) released by macrophages stimulates fibroblasts to deposit ECM molecules in the surrounding tissues [58]. miRNAs modulate both Smad and non-Smad dependent signaling pathways of TGF-β by targeting the key molecules that mediate transcription of ECM genes and TGFβ signaling [59,60]. Therefore, miRNAs can regulate collagen synthesis and other molecules involved in the progression of cardiac fibrosis. Moreover, several miRNAs have been investigated as biomarkers of LVR after MI [61].

We showed that none of the investigated miRNAs are differentially expressed and cannot differentiate between LVR and non-LVR patients. Interestingly, all plasma miRNA levels correlate to EF changes in non-LVR patients but not in LVR ones, while the changes in EDV are not correlated to any of the miRNAs. Of note, all three miRNAs are excellent MI discriminators, the performances of which are influenced by DM status and the level of plasma Hb.

miR-21 promotes cardiac fibrosis, hypertrophy, and angiogenesis and reduces apoptosis and inflammation [21,22,23,62,63,64,65,66,67]. Although miR-21 is one of the most investigated miRNAs (in five human trials) as a putative predictor of cardiac remodeling after MI, its value is not well established. Multiple variables might explain the heterogeneity of these results. In the Zile and Grabmayer studies, the cohort size is relatively small, LVR is defined as the absolute change of EDV (based on either echocardiography or CMR studies), and the follow-up period is rather short (three to six months); in these studies, miR-21 did not correlate with LVR parameters [68,69]. Dubois and Liu analyzed larger cohorts, defined LVR as an increase of at least 20% in EDV (measured by echocardiography), and had a good follow-up interval of one-year post-MI, but sampled blood late, at discharge; in these studies, miR-21 correlated with LVR parameters [18,70]. The study led by Gao and his colleagues suggests that miR-21 could be used as a post-MI survival predictor. It comprised a rather large number of patients, with blood sampling on admission, but the endpoint was death within 30 days, with no data on LVR parameters [71].

miR-150 protects against MI-induced fibrosis, and its prognostic use has been evaluated in four human trials [72]. Similarly, as for miR-21, the four studies for miR-150 are very heterogeneous in terms of cohort sizes, LVR definition, biological material of choice (plasma, serum), the timing of collection/follow-up, and endpoint evaluation; none overlap with our choices. Although Karakas et al. investigated a large number of patients, almost 70% of them had a history of MI, which is an exclusion criterion in most of the studies, including ours [73]. Devaux et al. used blood samples collected at discharge and echocardiography to assess LVR but modified the parameter used to evaluate cardiac remodeling: the absolute change of EDV in the first study is too indiscriminate, while the wall motion score index (in the second study) is too observer-dependent [74,75]. In addition, almost half of the patients did not receive any revascularization therapy (since it was technically unavailable at the moment and patient evolution was favorable), which might have significantly influenced the progression of LVR. The definition of LVR (EF ≤ 35%) and non-LVR (EF > 50%) proposed by Lin et al. leaves a substantial gap among patients with EF between 35 and 50%, which might also show signs and symptoms of HF [16].

While many reports support the role of miR-101 as protective against adverse cardiac remodeling, only one human trial confirmed its prognostic value [15,75,76,77]. The patients at risk for altered LV contractility show a combination of low miR-101/miR-150 and high miR-16/miR-27a plasma levels at discharge, while our evaluation was based on plasma samples at admission.

Many factors could account for the lack of concordance regarding the role of these miRNAs as LVR predictors, starting with the cohorts’ sample size, time of blood collection, detection and data normalization methods, and LVR definition. Our data indicate that patients’ comorbidities like DM and paraclinical parameters like Hb levels and erythrocyte numbers are potential confounders and could significantly influence the expression levels of all three miRNAs and their statistical association with ventricular remodeling. In this respect, a major limitation of our study (besides the modest size of the cohort analyzed) is the lack of an in-depth analysis of the impact of medication (like statins and antiplatelet drugs) due to the rather low adherence of our patients to the physicians’ indications [78,79].

## 5. Conclusions

Plasma miR-21, miR-101, and miR-150 fail as early (day of admission) biomarkers for LVR, despite being excellent discriminators of MI. Our analysis shows that the plasma levels of these miRNAs are strongly influenced by the patient’s diabetic status and hemoglobin levels. Future studies on much larger cohorts are needed to globally and comprehensively evaluate the impact of clinical and paraclinical confounding factors on the statistical association of miRNA to MI in general and to ventricular remodeling phenomena in particular.

## Figures and Tables

**Table 1:** Demographic and clinical features of STEMI patients.

Characteristics	All (n = 105)
Age, years	60.83 ± 12.9
Female, n (%)	29 (27.62)
**Cardiovascular history/risk factors, n (%)**	
Hypertension	75 (71.43)
Hypercholesterolemia	23 (11.90)
Current smoker	53 (50.48)
Obesity	31 (29.52)
Diabetes mellitus	24 (22.86)
**Presentation**	
Peak CK-MB (U/L)	108.97
Time from symptoms onset to reperfusion (hours)	6.34 ± 3.37
In-hospital death, n (%)	7 (6.67)
Thrombolytic, n (%)	21 (20)
**Type of infarction, n (%)**	
Anterior	49 (46.67)
Inferior	51 (48.57)
Other	5 (4.76)
**Medication, n (%)**	
Clopidogrel	52 (49.52)
Ticagrelor	46 (43.81)
Aspirin	98 (93.33)
Statin	98 (93.33)
ACEi/ARB	68 (64.76)
Betablocker	80 (76.19)
Aldosterone receptor antagonist	82 (78.10)
Nitrate	19 (18.10)

ACEi = Angiotensin-converting enzyme inhibitors; ARB = Angiotensin receptor blockers.

**Table 2:** Demographic and clinical features of the follow-up cohort of STEMI patients.

Characteristics	All (n = 43)	LVR (n = 14)	non-LVR (n = 29)	*p*
Age, years	57.81 ± 11.73	62.31 ± 11.32	56.6 ± 11.88	0.3545 *
Female, n (%)	12 (27.91)	3 (21.43)	9 (31.03)	0.509 **
**Cardiovascular history/risk factors, n (%)**				
Hypertension	33 (76.74)	12 (85.71)	21 (72.41)	0.332 **
Diabetes mellitus	8 (18.60)	2 (14.29)	6 (20.69)	0.61 **
Hypercholesterolemia	11 (28.58)	2 (14.29)	9 (31.03)	0.238 **
Current smoker	24 (55.81)	5 (35.71)	19 (65.52)	0.066 **
Obesity	16 (37.21)	7 (50.0)	9 (31.03)	0.226 **
Anemia: Hb < 13.5 g/dL (men), <12 g/dL (women)	7 (16.28)	4 (28.57)	3 (10.34)	0.128 *
**Type of infarction, n (%)**				
Anterior	23 (53.49)	8 (57.14)	15 (51.72)	0.741 **
Inferior	19 (44.19)	6 (42.86)	13 (44.83)	0.904 **
Other	1 (2.33)	0	1 (3.45)	
**Echo parameters**				
Average change EF (%)	5.35	−10.32	12.92	0.0003 *
Average change EDV (%)	3.77	26.65	−7.28	<0.0001 *
**Medication, n (%)**				
Clopidogrel	17 (39.53)	7 (50)	10 (34.48)	0.327 **
Ticagrelor	26 (60.47)	7 (50)	19 (65.52)	0.327 **
Aspirin	43 (100)	14 (100)	29 (100)	-
Statin	43 (100)	14 (100)	29 (100)	-
ACEi/ARB	33 (76.74)	9 (64.29)	24 (82.76)	0.180 **
Betablocker	37 (86.05)	11 (78.57)	26 (89.66)	0.328 **
Aldosterone receptor antagonist	36 (83.72)	11 (78.57)	25 (86.21)	0.522 **
Nitrate	8 (18.60)	4 (28.57)	4 (13.79)	0.242 **

* Unpaired two-tailed *t*-test with Welch correction; ** two-tailed Z test.

**Table 3:** Fold change and ROC analysis of normalized miRNA plasma values in STEMI patients vs. control group.

STEMI vs. Control			
	FC (*p* *)	ROC Analysis	
		AUC (%)	*p* **
**miR-101**	51.5 (0.003)	0.810	<0.0001
**miR-150**	3.7 (0.009)	0.780	0.0002
**miR-21**	11.7 (<0.0001)	0.866	<0.0001

* Unpaired heteroscedastic Student *t*-test with Welch’s correction; ** Wilson/Brown method.

**Table 4:** Correlation parameters (Pearson coefficient and *p*-values) between plasma miRNAs and clinical parameters in the STEMI patients’ group.

Spearman Coefficient (*p*)	DM	Erythrocytes	Hemoglobin	Hematocrit
**miR-101**	0.241 (0.013)	−0.345 (0.0003)	−0.258 (0.008)	−0.300 (0.002)
**miR-150**	0.352 (0.0002)	−0.193 (0.048)	−0.256 (0.008)	−0.182 (0.063)
**miR-21**	0.275 (0.004)	−0.135 (0.168)	−0.055 (0.575)	−0.108 (0.274)

**Table 5:** Fold change (vs. controls) and ROC analysis of normalized miRNA plasma values in STEMI patients in the three Hb subgroups.

	Hb < 13 g/dL		Hb: 13–15 g/dL		Hb > 15 g/dL	
	FC (*p* *)	ROC Analysis	FC (*p* *)	ROC Analysis	FC (*p* *)	ROC Analysis
		AUC % *(p* **)		AUC % *(p* **)		AUC % *(p* **)
**miR-101**	2.86 (0.047)	0.722 (0.025)	4.39 (0.0035)	0.819 (<0.0001)	5.84 (0.0013)	0.845 (<0.0001)
**miR-150**	1.90 (0.161)	0.660 (0.106)	3.89 (0.0007)	0.799 (0.0002)	4.77 (0.0002)	0.816 (0.0004)
**miR-21**	8.67 (0.0003)	0.830 (0.0009)	11.94 (<0.0001)	0.9071 (<0.0001)	13.77 (<0.0001)	0.912 (<0.0001)

* Unpaired heteroscedastic Student *t*-test with Welch’s correction; ** Wilson/Brown method.

**Table 6:** Fold change and ROC analysis of normalized miRNA plasma values in LVR vs. non-LVR patients.

		miR-101	miR-150	miR-21
**LVR vs. non-LVR**	**FC** (*p* *)	1.77 (0.095)	1.23 (0.547)	1.65 (0.189)
	**AUC** (*p* **)	0.667 (0.078)	0.547 (0.622)	0.631 (0.169)
**EF change %** **(LVR patients)**	**Spearman coefficient (*p*)**	0.374 (0.764)	−0.093 (0.274)	0.177 (0.912)
**EF change %** **(non-LVR patients)**	**Spearman coefficient (*p*)**	−0.514 (0.004)	−0.556 (0.003)	−0.509 (0.005)
**EDV change %** **(LVR patients)**	**Spearman coefficient (*p*)**	0.117 (0.690)	0.202 (0.485)	0.209 (0.470)
**EDV change %** **(non-LVR patients)**	**Spearman coefficient (*p*)**	0.036 (0.851)	0.223 (0.224)	−0.020 (0.919)

* Unpaired heteroscedastic Student *t*-test with Welch’s correction; ** Wilson/Brown method.

**Table 7:** Demographic and clinical features of MI patients with and without diabetes.

Characteristics	Diabetic (n = 24)	Non-Diabetic (n = 81)	*p*-Value (Z Test)
Age, years	63.63 ± 10.00	60.00 ± 13.59	0.159 *
Female, n (%)	10 (41.67)	19 (23.46)	0.081 **
**Cardiovascular history/risk factors, n (%)**			
Hypertension	21 (87.50)	54 (66.67)	0.048 **
Hypercholesterolemia	2 (8.33)	21 (25.93)	0.067 **
Current smoker	10 (41.67)	43 (53.09)	0.327 **
Obesity	9 (37.5)	22 (27.16)	0.327 **
**Presentation**			
Peak CK-MB (U/L)	88.29	114.68	0.394 *
**Type of infarction, n (%)**			
anterior	14 (58.33)	35 (43.21)	0.194 **
inferior	10 (41.67)	41 (50.62)	0.441 **
other		5 (6.17)	
**In-hospital death, n (%)**	5 (20.83)	2 (2.47)	0.0015 **

* Unpaired heteroscedastic Student *t*-test with Welch’s correction; ** two-tailed Z test.

**Table 8:** Fold change (FC) and area under curve (AUC) analysis in diabetic STEMI, non-diabetic STEMI, and controls.

	Diabetic vs. Non-Diabetic MI		Diabetic MI vs. Controls		Non-Diabetic MI vs. Controls	
	FC (*p* *)	ROC Analysis	FC (*p* *)	ROC Analysis	FC (*p* *)	ROC Analysis
		AUC, % (*p* **)		AUC, % (*p* **)		AUC, % (*p* **)
**miR-101**	0.56 (0.016)	0.666 (0.014)	2.84 (0.037)	0.7402 (0.0095)	5.05 (0.0002)	0.8308 (<0.0001)
**miR-150**	0.35 (0.001)	0.742 (0.0003)	1.62 (0.251)	0.639 (0.1315)	4.64 (0.0017)	0.8221 (<0.0001)
**miR-21**	0.50 (0.010)	0.6893 (0.0050)	17.61 (0.0003)	0.8186 (0.0006)	13.78 (<0.0001)	0.9179 (<0.0001)

* Student *t*-test with Welch’s correction; ** Wilson/Brown method.

## Data Availability

The data presented in this study are available in Supplementary Materials.

## Supplementary Materials

The following supporting information can be downloaded at https://www.mdpi.com/article/10.3390/biomedicines11102738/s1, Table S1: MicroRNAs and clinical data of the patients; Table S2: MicroRNAs and clinical data of the patients in the follow-up group.
